# Qualitative investigation of targets for and barriers to interventions to prevent psychosis relapse

**DOI:** 10.1186/1471-244X-14-201

**Published:** 2014-07-16

**Authors:** Emily Eisner, Christine Barrowclough, Fiona Lobban, Richard Drake

**Affiliations:** 1 Clinical Psychology Department, Zochonis Building (2nd Floor), University of Manchester, Oxford Road, Manchester M13 9PL, UK; 2 Division of Health Research, Spectrum Centre for Mental Health Research, Furness building, University of Lancaster, Lancaster LA1 4YW, UK; 3 Institute of Brain Behaviour and Mental Health, Jean McFarlane Building, University of Manchester, Oxford Road, Manchester M13 9PL, UK

**Keywords:** Relapse, Psychosis, Schizophrenia, Early signs, Basic symptoms, Prodrome, Intervention, Qualitative

## Abstract

**Background:**

Early signs based relapse prevention interventions for psychosis show promise. In order to examine how they might be improved we sought to better understand the early relapse process, service users’ abilities to identify early signs, and any potential facilitators and barriers to early signs interventions.

**Methods:**

Data from in-depth interviews with a convenience sample of service users with psychosis varying in gender, age, duration of mental health problems, and time since last relapse were analysed using a thematic approach. Interview transcripts were coded inductively and relationships between emerging themes were examined by the research team to provide a thorough synthesis of the data.

**Results:**

Three central themes emerged from the analysis: 1) *recognising risk factors* (how risk factors were identified and linked to relapse, and reactions to such risk factors); 2) *identifying early signs* (issues related to both recognising and recalling signs of relapse); 3) *reacting to deterioration* (participants’ thoughts and feelings in response to early signs, including help seeking and its challenges).

**Conclusions:**

There was considerable variation in the attention participants had paid to pre-relapse signs, the ease with which they were able to recall them, and their reactions to them. For many, there were substantial barriers to help seeking from services. A family or friend confidant was an important means of assistance, although the supportive presence of significant others was not always available. Based on these results, a number of recommendations about facilitating service users’ recognition of early signs and targeting potential accelerants of relapse are made.

## Background

Since relapses of psychosis are prevalent [1] and problematic [2-4], effective relapse prevention interventions are needed. Early signs based relapse prevention interventions work on the premise that timely prediction of relapses will allow preventative action to be taken, minimising the chance of full relapse. Service users are encouraged to monitor their own mental state and if early signs of relapse emerge (e.g. insomnia, anxiety, dysphoria, attenuated psychotic symptoms) to act upon a pre-agreed, concrete plan (e.g. increased medication, increased support, stress reduction). Such interventions show promise but could be further improved [5]. Qualitative methods are suited to exploring how an intervention works and potential barriers to its effective implementation [6], complementing quantitative approaches [5] that necessarily impose a preconceived structure on such experiences and may overlook unexpected, important findings. We therefore used thematic analysis of in-depth interviews to better understand the early phase of relapse, service users’ abilities to identify early signs, and any other potential facilitators of and barriers to early signs interventions.

Although a number of previous papers have discussed such facilitators and barriers [7-12] the evidence is largely anecdotal [10-12]. The few existing qualitative studies [7-9] comprised interviews or focus groups with no more than ten participants from any one group (service users, staff or carers) and either conducted fairly superficial analyses (Alceste textual analysis [9]) or are unsubstantiated by quotations from participants [7,8]. Nevertheless, these studies make useful observations on the role of family members in early signs interventions [7,9], the need for therapeutic alliance with clinicians [8,9], the value of idiosyncratic early signs [7,9] and the challenges posed by negative symptoms or cognitive difficulties [8]. One further qualitative study [13] used a rigorous interpretative interactionism approach to examine how services users develop the ability to detect early signs of relapse. It outlined a shift from responding reactively to emotional distress to responding pro-actively. Unlike the current study it did not examine any one episode in detail but looked for patterns developing over successive episodes.

We conducted a thorough, transparent analysis of in-depth interviews with a diverse, relatively large sample of services users who had recently experienced a relapse of psychosis. We retrospectively examined deterioration from initial triggers to the onset of relapse itself and obtained narratives of this process and reflections on how easy or otherwise it was to recount, supplemented by direct observations during the interviews.

The study aimed to examine how the early signs approach to relapse prevention might be improved; to this end service users’ experiences and reactions early in the relapse process were explored. Accordingly we have made a number of clinical recommendations about facilitating service users’ recognition of early signs and targeting potential accelerants of relapse, as well as suggestions for future research.

## Methods

Ethical approval was obtained from the Liverpool Central research ethics committee (ref: 12/NW/0091). The study is reported in line with recommendations outlined in the COREQ checklist [14].

### Sampling and recruitment

Participants were recruited from three NHS Mental Health Trusts in the North of England between May and November 2012. Inclusion criteria were: aged over 18 years; a current, primary clinical diagnosis of non-affective psychotic disorder (DSM-IV) [15]; at least one episode of acute psychosis in the past 6 months requiring admission to crisis team or inpatient unit; currently prescribed antipsychotic medication; no illicit drug use or alcohol abuse or dependence during the pre-relapse period; able to give informed consent.

We aimed to recruit 20 to 25 service users to capture a diversity of experiences. Recruitment stopped after 23 interviews as a consistency was emerging in the themes across the participants. The convenience sample had a good mix of key variables (age, gender, ethnicity, number of psychosis episodes, inter-episode symptom level and length of time since admission) so more purposive sampling was unnecessary. A summary of clinical and demographic characteristics is given in Table 1 and basic descriptions of individual participants are given in Table 2.

**Table 1:** Summary of clinical and demographic characteristics of the study sample (n = 23)

	**Frequency**	**Percentage**
Diagnosis		
Schizophrenia	17	(73.9)
Schizoaffective disorder	6	(26.1)
Number of psychosis episodes		
Two	4	(17.4)
Three	12	(52.2)
Four or more	7	(30.4)
Level of inter-episode symptoms		
None	6	(26.1)
Low level	15	(65.2)
High level	2	(8.7)
When discharged from hospital or crisis team, relative to interview		
Not discharged (hospital)	7	(30.4)
<1 month ago (hospital) and/or not discharged (from crisis team)	10	(43.5)
>1 month ago (hospital) and/or < 1 month ago (from crisis team)	2	(8.7)
>1 month ago (hospital and/or crisis team)	4	(17.4)
PANSS positive sub-scale score, mean (SD)	16.87	(5.4)
Age, mean (SD)	38.4	(14.0)
Gender, n male	11	(47.8)
Ethnic origin		
Asian or Asian British	3	(13.0)
Black or Black British	3	(13.0)
White British	16	(69.6)
Other ethnic group	1	(4.3)
Education		
None or primary only	3	(13.0)
Secondary	6	(26.1)
Further (e.g. A levels or Diploma)	7	(30.4)
Higher (e.g. bachelor’s degree)	7	(30.4)
Employment		
Retired	3	(13.0)
Home duties	1	(4.3)
Unemployed	19	(82.6)
Living arrangement		
Family or partner	9	(39.1)
Alone	11	(47.8)
Shared/supported accommodation	3	(13.0)
Level of family or carer contact		
None	8	(34.8)
Low	8	(34.8)
High	7	(30.4)

*Note:* Values are frequency (percentage) unless otherwise specified.

**Table 2:** Basic characteristics of individual participants

**Participant**	**Gender**	**Age**	**Number of psychosis episodes**
P02	Female	24	2
P03	Female	48	≥4
P04	Female	62	3
P05	Female	64	2
P06	Male	19	3
P08	Male	57	≥4
P10	Male	43	3
P12	Male	32	3
P14	Male	51	≥4
P18	Female	47	≥4
P19	Female	22	3
P20	Female	24	3
P21	Female	27	3
P22	Female	39	≥4
P25	Female	39	3
P26	Female	33	3
P29	Male	37	≥4
P30	Male	24	2
P32	Female	33	2
P34	Male	24	3
P35	Male	64	≥4
P36	Male	35	3
P37	Male	34	3

Clinicians referred patients they judged capacitous and suitable. The way in which the research was presented to potential interviewees may have affected their decision to participate and the content of their narratives. We explained that we were interested in their experiences in the few months before their recent admission, especially any changes that they noticed at an early stage. We emphasised that their decision whether to participate would not affect their clinical care and that, aside from urgent risk information, nothing from the interviews would be passed to the clinical team without the participant’s consent. Participants knew that they had been selected due to their diagnosis of psychosis and several agreed to take part, knowing this, despite not agreeing with their diagnosis. They were offered shopping vouchers (£15), which may have influenced their decision to participate but is unlikely to have affected the content of the interviews.

### Qualitative interviews

Interviews took place during a one-off meeting at the participant’s home or an NHS service, with all but two conducted by the first author (EE, a female PhD researcher with a Master of Research degree which included a qualitative methods component) (CB and RD conducted one interview each). All interviewers identified themselves as researchers and not members of any clinical team. In four cases a family member (P06, P37, P21) or carer (P35) was also present during the interview at the participant’s request. Interviews lasted 30 to 90 minutes, were audio recorded and later transcribed. For logistical reasons, we were unable to consult participants for feedback on data transcription or analysis.

Based on a topic guide (available on request), developed by the research team in consultation with two pilot participants, the interviewer used open questions to explore events, feelings and experiences prior to the participant’s most recent episode of psychosis. Topics used to promote discussion of this period included: participants’ daily routines, their relationships with others, how they felt in themselves and how things around them seemed. The topic guide was used flexibly to guide the interview, the aim being for the researcher to gain an in-depth understanding of the participant’s experiences rather than to obtain answers to specific questions. Probe questions were used where applicable to prompt further elaboration. Immediately after the interview, the interviewer made a note of any possible themes, additions to the topic guide or relevant contextual details.

### Analysis

Analysis of the qualitative data was conducted by a PhD researcher (EE), a clinical psychologist (CB) and a psychiatrist (RD), in consultation with a clinical psychologist with experience in qualitative research (FL). Our thematic analysis followed a social constructionist approach [16], with interview transcripts coded inductively and relationships between emerging themes examined to give a thorough synthesis of the data. The initial coding scheme was developed by the whole research team, based on reading, discussion and preliminary coding of the first three interviews. The remaining transcripts were then systematically coded by EE, managing the process using the NVivo software package [17]. The coding scheme was constantly updated as it was applied to further interviews. Emerging themes were discussed and refined within the wider team to ensure transparency and rigour, with attention paid to why ‘deviant cases’ did not fit the emerging theory. When initial coding of all interview transcripts was completed, a final coding scheme was agreed and all transcripts were re-coded to ensure consistent coding of the whole dataset. All ideas, insights, discussions and decisions during data collection and analysis were documented. Together with the post-interview memos, these notes provide an audit trail giving a transparent account of the process.

The mix of professional backgrounds within the research team promoted consideration of a variety of interpretations after thorough scrutiny of the data. Nevertheless, we inevitably brought certain assumptions and expectations to the interview and analysis process. In conducting a study aiming to inform relapse prevention, we assumed that relapse is something negative that should be prevented. Based on the research literature, we anticipated that service users would experience changes early on in their deterioration that may be useful early signs of relapse; a key focus of the interviews was to elicit descriptions of and reflections on such changes. Finally, our reading of the wider psychosis literature, including medical (e.g. ICD-10) [18] and psychological [19] models of psychosis, is likely to have influenced data interpretation.

## Results and discussion

Some participants were very reflective as they spoke about their experiences prior to relapse, clearly having already given the topic a lot of thought. Others were notably unreflective, suggesting that they had rarely, if ever, considered it before. Communication was influenced by language (all were fluent English speakers but for three this was not their first language) and apparently by phenomena such as thought disorder.

**Figure 1:**
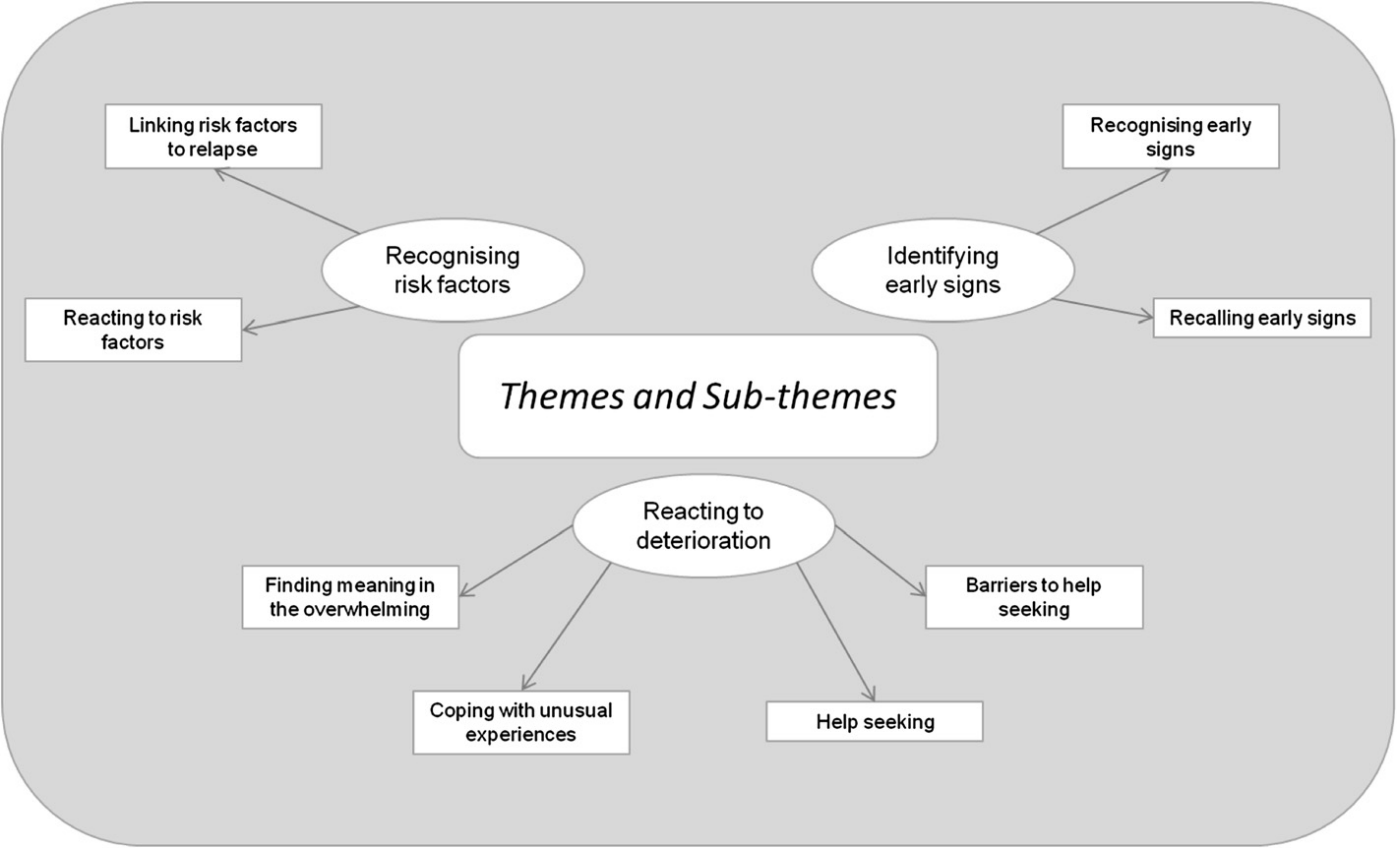
Themes and sub-themes emerging from the qualitative analysis.

Three themes relating to service users’ experiences in the early stages of relapse emerged from the analysis of the data (see Figure 1). *Recognising risk factors* captures participants’ accounts of significant events or stressors that occurred during the few months prior to their recent relapse, whether they saw them as risk factors for relapse, and their reactions to these experiences. Secondly, service users outlined changes in their internal experiences during the earliest phase of their recent deterioration in mental health that may be useful as early signs of relapse. Whilst the nature of these changes will be covered in a companion paper, the ease with which participants were able to recognise and recall them emerged as a strong theme in the current study, labelled *identifying early signs*. Thirdly, *reacting to deterioration* depicts participants’ thoughts and behaviours in response to these subjective changes.

### Recognising risk factors

#### Linking risk factors to relapse

As might be expected from existing literature, interpersonal stress [20], life events [21,22], isolation [1,23] and medication reduction [1,24] were reported by many participants as precursors to their recent deterioration. Although easily identified in retrospect, not everyone linked them to their subsequent relapse. Some did; for example, one participant described a gradual progression from her stressful interactions with her neighbour to her later fears that he, and eventually everyone, was going to kill her.

“It wasn’t until when he started complaining, and then it was like everyone was against me because I couldn’t get anywhere with him, but it was quite rational up to – and then it went irrational” [P04].

Participants who made such associations tended to meet criteria proposed for ‘insight’, for example at least partially agreeing with their diagnosis [25-27]. This is worth bearing in mind clinically when considering whether an individual would currently benefit from an early signs intervention. Since insight increases as symptoms resolve following an acute episode [28], the timing of such an intervention may be a key to its success.

On the whole, participants did not readily link *positive* life events, such as starting a new job or college course, to their subsequent deterioration, despite evidence that they are risk factors [21]. Nevertheless, one participant developed her view as the interview progressed until she reached the conclusion that anticipation of a new job may have been linked to her relapse.

“the [*name of city where first episode occurred*] was completely stress, whereas this time round I don’t know what it was, maybe just the anticipation of going into an office environment” [P02].

The reluctance to acknowledge positive life events as risk factors may reflect the potential cognitive challenge: if even positive life events are risky, how do I improve my life? This paradox may need to be worked through cognitively as part of the intervention. Service users may be encouraged by the idea that an early warning signs approach provides them with tools for dealing with the challenge. By learning to recognise early signs of deterioration they can make progress whilst maintaining a sense of control of their mental health, rather than avoiding positive life events.

Some linked isolation to relapse: some simply made a chronological link, whereas others’ descriptions hinted at three potential mechanisms by which these experiences may impact psychosis: the absence of opportunities for reality testing (see *finding meaning in the overwhelming* sub-theme), the lack of assistance in seeking professional help (see *help seeking* sub-theme) and the stress of coping alone (see quote below).

“It got worse when my mum went away from me ‘cos she’s always with me. And I used to tell her if anything bothered me and if I tell somebody else they wouldn’t understand what was happening.” [P21].

This is consistent with the finding that high availability of attachment protects against relapse after a life event [29].

Unlike other risk factors, reducing or stopping medication was viewed by almost everyone who mentioned it as a trigger for their deterioration. Only one participant said he felt better after he had stopped the medication (although other content of the interview suggested that he got more psychotic).

“me I’m fine. Feeling good. Stop the medication, feeling very good. So take medication, so me have, make me paranoid, make me scared” [P12].

Otherwise, the closer in time the two events, the more easily participants made an association. Two participants who stopped the medication after the psychotic symptoms had begun to get worse saw this step as accelerating their deterioration.

“I kidded myself I could get by without it… I became more and more psychotic” [P35].

Similarly, a Taiwanese qualitative study [30] found that service users often began to value medication through trial and error. For those who stop or reduce medication several months prior to relapse, discussion of the protective role of medication in the context of stress-vulnerability may help them to make the link. Though, as with any medication, the potential risks and benefits of long term antipsychotic use need to be carefully considered [31-33], the need to be all the more vigilant when it comes to life events or other risk factors after reducing or stopping medication should be emphasised during early signs intervention.

#### Reacting to risk factors

Participants tended to describe reactions to risk factors, including life events or interpersonal stressors, such as sadness, fear, and rumination.

“Well the main er thing is er my uncle passed away, March 29, so my mum went Pakistan and I was really missing my mum. And er I started getting depressed and stressed out… and I was really really sad and uncontrollable I was crying for no reason, just started from there” [P21].

“if I’d had a tricky day, perhaps problems with strangers or officialdom I – I had so much going on in my head trying to analyse it that I wouldn’t actually get any sleep” [P35].

The current early signs literature tends to characterise early signs (including changes in feelings) as stand-alone, spontaneous changes, so there may be a danger that emotional changes in response to life events and other stressors may be discounted. Including such changes in self-monitoring strategies, understandable reactions though they may be, may improve relapse prediction.

Furthermore, the negative emotions may themselves be potential targets for therapy. Not only are they unpleasant in their own right, they may contribute to the development of psychosis. Two existing cognitive models of psychosis relapse [34,35] propose that dysphoric early signs (e.g. anxiety, depression, arousal, insomnia) are responses to internal or external events resembling the early stages of previous episodes and that, as such, they can be causal factors rather than just inert epiphenomena of imminent relapse. To our knowledge, only one study to date [36] has used a targeted psychological therapy (cognitive behavioural therapy) to directly address these states. Relapse rates were significantly reduced in those receiving the intervention, but this result can only be considered preliminary due to methodological limitations [5].

When participants in the current study had reduced their medication after feeling well for some time, they often expressed anticipatory anxiety or regret about having done so.

“I was worried like cause you know the medication makes you go to sleep it’s starts causing drowsiness so I was like, well I’ve been on this for three years, erm how is this gonna really affect me” [P02].

“…I said ‘look I’m well now, can I change it?’”; “That was the worst thing I could have done.” [P03].

That progressing from feeling well to a dose reduction could lead to deterioration may disturb some individuals; they may find that early signs interventions enable them to reduce medication whilst managing their anxiety by creating a sense of being in control.

### Identifying early signs

#### Recognising early signs

Details of the changes in experience identified as early signs of deterioration will be described in a companion paper along with quantitative information regarding their frequency in this sample. Participants reflected on how easy or otherwise it was to identify early signs, highlighting a number of potential challenges, discussed below.

One participant’s relapse was simply an exacerbation of the voices that he had heard for several decades and he could identify no early signs of this deterioration. Nevertheless, others with persistent symptoms were able to recognise changes preceding an exacerbation. For some this awareness had taken some time to develop but was then seen as an asset.

“erm to be honest erm until this kind of episode I did lack insight - erm and I knew that these things were changing but I couldn’t see the connection… erm but this time, kind of with hindsight and just bit more awareness really so I’m hoping that will kind of aid me in the future really” [P19].

Several participants commented that their willingness to talk about the process of relapse had increased over time, implying that they would previously have struggled to discuss it. This is consistent with a transition from “sealing over” to “integration” [37,38] of the episode into a positive self-concept. Given that sealing over is associated with poor engagement with services [39], service users in this group may find identifying early signs particularly difficult. For some the transition to a more integrative recovery style might be a necessary precursor to participating in an early signs intervention.

The speed at which episodes occurred could pose a challenge. Some participants experienced the onset of psychotic symptoms as *abrupt*, often using language that highlighted the impact.

“I were decorating one day and then it were like, it were like. It weren’t my head exploding or anything. It were just like ‘bang’ – there were like these voices coming from everywhere around” [P36].

Some identified no change in the run up to the psychotic symptoms, for example emphasising that they maintained their normal routine in the run up to their deterioration and that their perceptions were unchanged.

“Oh just doing normal things like everybody else.”; “everything was just usual.” [P03].

Nevertheless, as the interviews progressed several of these participants began to identify early signs retrospectively. This was also the case for those who identified having a very *gradual* deterioration as a potential challenge, since they were unable to detect any early signs at the time (although significant others sometimes could).

“I don’t think if erm if I was by myself cos most of the time all the time my family was there to help me er, I don’t think I would have noticed things changing that gradually” [P34].

“it’s very difficult for me to give you a timeline… ‘cos it’s very difficult to notice your own malfunctioning downwards into a spiral… I’m probably learning more of it by talking to you” [P04].

As the second quote illustrates, the interview was a catalyst in working out what had happened, with questioning and reflection from a third party appearing to facilitate this process. This may also be why some found the assistance of family or close friends invaluable:

“Would it be possible for my mum to come in? ‘Cos my mum monitors me the most” [P06].

Although family members can clearly play an important role, service users’ own ability to identify early signs, given appropriate support, should not be underestimated. Unlike carers, service users have access to *subjective* as well as objective changes [7]. Furthermore, some participants recognised a pattern of early signs that had occurred prior to several previous episodes with minimal assistance. Others who did not spontaneously make comparisons with previous episodes often acknowledged that there was a pattern when asked by the interviewer.

“Yeah there must be a pattern to it… Like not getting any sleep, like two hours a night” [P03].

#### Recalling early signs

Although all participants had experienced their most recent relapse within the last 6 months, some struggled to recall the early stages of this deterioration. Nonetheless most of these people were able to remember at least some aspects of this period when guided by the interviewer’s open questions. There was no discernible relationship between difficulty remembering and how long ago the deterioration occurred. Nevertheless, practitioners negotiating an early signs intervention might make this decision on a case by case basis, taking into account the service user’s current ability to remember the recent episode and their current level of insight. They should also set aside plenty of time for the initial identification of early signs, since we found that the amount of time invested in the interview appeared to affect the degree of recall, with more time resulting in more elaborate and comprehensive descriptions of early warning signs.

Several people able to construct a rough timeline for their deterioration still found providing details of when or for how long particular experiences had occurred difficult.

“it’s just that I wish I could remember more so that I could help you… the dates… well obviously if you slide into your imagination, the dates will disappear” [P04].

The interviewer tried to help participants anchor their experiences by linking them to key calendar events (e.g. Christmas) and sketched out a written timeline with participants. Some appeared to find this a useful reference whereas others rarely referred to it. For some the struggle to remember took place within the context of wider cognitive difficulties.

“I can’t remember little things… it has to be a big massive thing to remember, do you understand me?… I can’t remember these little things what’s happening” [P29].

Conversely, one participant felt that the early stage of her deterioration *was* a big thing, and thus memorable.

“it’s like a big thing for me so usually I do remember it” [P20].

During the interviews, participants used various strategies to help them remember the process of their deterioration. Several participants had already given the topic some thought in readiness for the interview and came with handwritten notes as a cue. One participant referred to emails and letters written at the time of interest; another used her diary as a prompt during the interview. Given that participants already used a variety of strategies, it seems important for interviewers to be flexible and make use of them.

### Reacting to deterioration

#### Finding meaning in the overwhelming

Participants described reactions to the changes they experienced, including both thoughts (this section) and behaviours (see *coping with unusual experiences* and *help seeking* sub-themes). Some initially said that they felt in control of their experiences and as a result were not concerned about the idea of having another episode in future. Nevertheless, at other points in the interview, the same participant would describe having become overwhelmed by her experiences over time.

“I was walking through a patch of green land… and I just thought ‘I’ve had enough of all this’, you know ‘I’m tired’ and what have you and I just laid down on the grass and then I just let it all out really, everything that’d been building up over the months. And I ended up shouting out lots of things” [P32].

Becoming overwhelmed by increasing unusual experiences was a critical moment in the process of deterioration for some, often as their capacity to normalise them failed.

“I must admit, you know I thought it was just because I was tired that I was falling asleep but… towards the end of it I realised, no it’s not that, it’s everything electrical in my home.”; “I started going out and I became very observant and you know I was getting these dreams and it was all a bit too much for me to be honest, all in one go.”; “it all seemed a mess in my head. I couldn’t figure any of it out. I was like stuck in the middle of a tunnel and there was like loads of little tunnels off it… and they all seemed to lead to somewhere I didn’t want to be… it’s like being in a maze and I couldn’t get out” [P22].

These accounts are consistent with Garety and colleagues’ [19] model of psychosis development. According to the latter, the search for meaning is a crucial step in the transition from anomalous experiences (in response to a stressor) to full blown psychotic symptoms, with the attribution of an external cause being the pivotal moment in this process. If this is the case, *in vivo* interventions targeting these cognitions would be valuable. Few early signs interventions to date have directly addressed cognitions during the deterioration process, the exception being Gumley and colleagues’ [36,40] targeted cognitive behavioural therapy. In this case, beliefs and behaviours thought to be accelerating relapse were identified in order for alternative beliefs to be developed and reinforced through behaviour change. However, such an approach requires skilled therapists, which may limit availability.

In the current study, family and friends sometimes played a role in reinforcing normalising explanations during the search for meaning process.

“I haven’t realised that it’s something that someone else would see as a small thing, so I have mentioned it. And then like someone else has said to me like ‘oh you probably have put it down somewhere or something’, you know like or just say ‘you sound a bit weird’ or something, and sometimes I need that, you know, just to – and then I’ll think like ‘oh right’ you know [*laughs*]” [P20].

This is consistent with our earlier suggestion that one mechanism via which social isolation may increase the risk of relapse is that the absence of trusted others removes the potential for reality testing, and with the finding that social network size can be correlated with awareness of illness [41,19]. Not only is reinforcement of normalising explanations something that a care co-ordinator could assist with in the absence of other supportive relationships, they may be able to work with the service user and their family to strengthen and encourage existing cognitive strategies.

Finally, if the transition from feeling in control of one’s experiences to feeling overwhelmed by them is a key turning point in the process, it may be a useful warning of imminent relapse that service users should be encouraged to heed. Their monitoring checklist might include statements such as ‘it feels like things are getting on top of me’ and ‘I am spending time trying to work out why things seem different’.

#### Coping with unusual experiences

One of the aims of an early signs intervention is to promote adaptive coping strategies early on in the deterioration process in order to minimise the chance of a full relapse occurring. So, it is helpful to examine participants’ habitual coping behaviours in order to consider the extent to which adaptive strategies are already in place. Strikingly, many of the coping strategies described by the current sample involved some form of distraction or avoidance. For example, one participant reported using her sleep medication to enable her to sleep more than usual in order to avoid the stress of her unusual experiences.

“yeah, so I take er… I take Zopiclone and get in bed, I take em all the time, when I wake up I take another, when I wake up I take another” [P18].

This was often accompanied by comfort eating:

“It’s mainly sweet stuff really, I’ll just like, I think it’s just, it’s just like a comfort thing. I think it’s the same with staying in bed it’s just like I just want to be snuggled up and feel like protected so. It’s just anything that makes me feel a bit better really” [P20].

Other similar strategies included avoiding situations where symptoms were prominent or distracting oneself from the unusual experience by keeping busy or listening to music. The prevalence of avoidant coping strategies in the current study is consistent with previous findings that these are more common among individuals with psychosis than the general population [42]. It is possible that this relationship is influenced by worse neurocognition [43], higher neuroticism [44], more irregular sleep [45] and lower self-efficacy [46,47]. An early signs intervention may raise service users’ perceptions of their ability to cope by introducing specific techniques (e.g. monitoring, action planning) and a conceptual framework for systematising coping at various stages of deterioration. Future studies that aim to evaluate such an intervention should examine the role of self-efficacy and other potential mediators or moderators, particularly in relation to avoidant and maladaptive coping.

In terms of more active coping strategies, most participants mentioned help seeking (see separate sub-theme, below), and a number mentioned using medication: for example, greater adherence or taking extra medication in response to early signs of deterioration.

“‘Cos, erm, I notice it even if I miss a couple of my tablets, I’ll be like erm, ‘cos I’m really forgetful sometimes I forget to take them and then like I’ll be outside and I’ll just think oh like I need to take them ‘cos they’re like um, just like noises are annoying me” [P20].

One participant emphasised her use of stress reduction techniques prior to her first episode, and noted elsewhere in the interview that she had been careful to choose a low-stress job prior to her recent episode.

“I was doin everythin possible to combat stress… so I’d be jogging in the morning with [*name 4*], come back an get changed, have something to eat, breakfast, an then I’d walk into town to do the shopping … an I was reading so I was doin everythin that’ I’d done in uni to combat stress” [P02].

This was the only mention of a strategy directly addressing stress, despite a number of participants having described significant stressors prior to and during the early stages of their deterioration, which implies that an early signs intervention should include targeted stress reduction strategies.

Of course the fact that all those in the current study eventually relapsed may introduce a bias towards ineffective coping strategies. Future studies comparing coping strategies in those who do relapse and those who do not may be of value in the development of interventions [42].

#### Help seeking

Help seeking emerged as an important sub-theme in the interviews; almost all of the participants talked about experiences of seeking help, barriers to help seeking, or both. Since an early signs intervention would aim to promote earlier and more effective help seeking it is useful to examine what characterized help seeking at each stage in the deterioration process: *early* on, in response to early signs such as sleep difficulties, and *late* on in response to full psychotic symptoms.

Participants sought help from different sources at different stages. Those who sought help early on were most likely to talk to family or friends. One participant noted that, although she talked to her partner about the first signs of deterioration (e.g. noticing grass looking greener than usual), she tended not tell her CPN until later on when the psychotic symptoms became upsetting; this was, at least partly, due to the infrequency of contact with her CPN.

“Yeah I’ll mention it yeah erm, and like [*partner’s name*] will say like um ‘have you taken your meds’ and stuff like that… But um, I’d say it more to [*partner*] than to like my CPN because like I only see [*CPN*] every three weeks… So it’s a bit like, ‘cos it happens so quick, it’s like I don’t know, I’d feel weird phoning up and going like ‘oh I noticed some grass’ or something so [*smiles*]” [P20].

When participants talked to family or friends this often took the form of confiding rather than active help seeking. However, this confiding sometimes caused others to seek help on their behalf.

“as I say when, when I heard the music and I started to hear the voices and I rang [*friend’s name*]… So she got concerned and so she rang the crisis team” [P05].

This is consistent with lack of assistance in seeking help being a mechanism by which social isolation increases relapse risk. Likewise, before first presentation social isolation is associated with a longer duration of untreated psychosis [48], whereas family involvement in help seeking is associated with a shorter duration of untreated psychosis [49]. Table 1 shows that only a small proportion of the sample had high levels of contact with a family member or significant other, an almost identical proportion (30%) to that reported in a large randomised controlled trial with similar inclusion criteria to our own [50]. Availability of social support is something that clinicians should assess when conducting early signs interventions, perhaps focussing on lowering the threshold for contacting statutory or other services for those without a natural confiding relationship.

Those who sought help late in the deterioration process often did so chaotically and from non-health services such as the police. This typically occurred when the explanations (e.g. mental illness) and help (e.g. increased medication) offered by mental health services did not fit participants’ models of their experiences.

“there were many cases when I called the antisocial behaviour, when I called the police, when I called the local organisation like er council” [P30].

“all the time is when I called the police everyday, police no do nothing.” [P12].

“I texted my pastor a lot, I emailed him a lot with all the emotional stuff and so on, and he didn’t know what to do about it” [P04].

This indicates a need for close working between mental health services and other public services such as the police, who often play an important role in managing mental health crises in the community [51], despite often having inadequate training in this area [52]. A number of initiatives such as the Crisis Intervention Team model [53] in the US have sought to deal with this discrepancy by establishing core teams of police with specialist training in dealing with mental health crises. Such approaches may be relevant to early signs interventions, if only as a ‘plan B’.

#### Barriers to help seeking

Service users often encountered significant barriers when actively seeking help from primary and secondary health services, as exemplified by the following quote from an individual who was diligent in seeking help for clear psychotic symptoms but felt that she was often fobbed off, delaying effective treatment until deterioration had progressed.

“I went back to him and said look, I need something, you need to refer me to the crisis team and that’s when he said ok he’s gonna write the letter to the crisis team but he didn’t give me olanzapine because they had to assess me first to give me the medication”; “but because it kept – the auditory hallucinations were more prevailing an they were worse you know… from that point on I just went to the accident and emergency but they said they couldn’t do anything for me at all – one of the woman were just laughin basically, well not laughin-laughin but it’s just her reaction coz I was goin for the help, it was like she didn’t believe me… so I got sent back home an then I went back to the crisis team an they told me to go back to A & E an then I seen a consultant in the A & E department for psychologists an that’s when he gave me the olanzapine.”; “…it was about … yeah a week an a bit before I got them … an I started on 5 milligrams but it still got worse” [P02].

These individuals were all under the care of specialist mental health services at the time of deterioration. Barriers were reported by participants in all three Mental Health Trusts, in both rural and urban areas. Although it was not clear why these barriers were so abundant, there were some hints in participants’ narratives. Firstly, in some cases there appears to have been a lack of clarity as to the correct pathway of care and an incentive for services to avoid work so that service users were passed between services for some time before receiving any assistance. Secondly, there may have been a lack of interest or expertise among primary care health professionals (e.g. general practitioners) regarding risk factors and early signs of relapse of psychosis, and even regarding psychotic symptoms themselves. Finally, in some cases it may be that clinical teams became overstretched and preoccupied by immediate risk so they adopted the maladaptive strategy of only responding to crises. Service delays have been shown to contribute to the duration of untreated first episode psychosis [54-57] but little research has examined help seeking barriers in *relapse* of psychosis.

It is worth cautioning that another bias introduced by selecting service users who had all relapsed was towards service failure and delay. Nevertheless, it raises the question of whether it is ambitious to expect teams to assist service users in using an early warning signs approach, given that in some cases they fail to assist people who are actively seeking help and clearly psychotic; there is little point in being able to identify relapse early if help seeking is ineffective. However, emphasising the idea that the early signs approach makes it easier to identify relapse early, before it becomes more difficult to deal with, may be a significant motivation. It is likely to give clinical teams more tools for dealing with the relapse process, for example by providing a positive script of what to do when people show early signs of relapse; this may improve clinicians’ sense of their own efficacy. Furthermore, the focus on identifying and managing relapse early might help staff to think through the consequences of making help seeking difficult for service users. For some the difficulty accessing help became an additional source of stress, which had a notable impact on functioning and may have also accelerated relapse.

“when I was trying to get to my nurse and trying to get to [*housing association*], to get proper help, at that point I’d be phoning all morning, and then I wouldn’t eat, and I’d phone my pastor and I wouldn’t eat and I’d stop shopping and I’d stopped eating and I stopped cleaning simply because I hadn’t got the time and I’d just eat nothing” [P04].

A subset of participants neither sought help nor confided. Most of these individuals spontaneously discussed why that was. For example some simply found it difficult to articulate how they were feeling.

“you know when you talk to the doctors… and when you talk to other people about the experiences you’ve had – your illness – if it is an illness – it’s very hard to put into words how you actually feel” [P08].

This participant felt that cognitive problems and time constraints of appointments contributed to this difficulty. He also preferred not to disclose some of his more unusual experiences to avoid anticipated reactions.

“I never mention it to anybody, only you [*Interviewer’s name*]… Cos people’d think you’re round the bend, they’d think this one’s lost the plot” [P08].

This was echoed in others’ accounts and seemed to be particularly the case for a certain type of subtle, subjective experience described by some participants (e.g. shapes appearing distorted; colours seeming brighter). As will be described in a companion paper, these subtle subjective changes in service users’ experience of the world around them resemble ‘basic symptoms’, which are used in German psychiatry as early indicators of first episode of psychosis [58]. Participants were unlikely to seek help when they occurred, since they did not expect mental health professionals to understand them. These fears may be justified since these subtle subjective experiences are not yet widely used in the UK as early signs, despite some retrospective evidence that they increase prior to relapse [59].

Although further research is needed regarding the predictive value of basic symptom-like experiences [5], current recommendations suggest that early signs of relapse may be very idiosyncratic and, as such, any signs which appear to be part of an individual’s relapse pattern should be taken seriously. For this type of subtle experience to be usefully included in an early signs intervention, staff and family members must be made aware of them and taught to react appropriately when service users mention their occurrence. This is likely to encourage service users to be more open about them, facilitating appropriate help seeking. Describing them as understandable reactions to stress that others experience too was an approach which appeared to be acceptable to interview participants, even those who did not hold an illness model or agree with their diagnosis.

Strained relationships with significant others also hindered communication in some cases. One participant was living with her parents, in what seemed to be a household with high expressed emotion, and found help seeking difficult:

“there’s no maternal thing and we were arguing and I reverted back to teenager behaviour… that’s all they saw of me so they didn’t know what I’ve done in my past and the achievements I’ve achieved and the way I’ve self-supported myself. All they saw was just this teenage rebellious woman trying to communicate with her so called parents but verbally I couldn’t communicate” [P32].

In such cases Family Intervention [60], in which the expressed emotion in the family environment is a target for therapy, may be a useful adjunct to an early signs approach.

At least one participant had a general reluctance to disclose their experiences, e.g.:

“my mum did raise you know some concerns how I was feeling… but I wasn’t very open with her at times” [P19].

This may relate to individuals’ attachment styles, hypothesised to develop in response to childhood interpersonal experiences, to be generally stable over time and to influence adult psychosocial functioning [61]. Clinicians may benefit from being aware of service users’ attachment styles since avoidant attachment styles may pose a challenge for an early signs approach if they are not addressed.

## Conclusions

There was considerable variation in the attention participants had paid to risk factors and early signs of relapse, the ease with which they were able to recall them, and their reactions to them. Guided by the interviewer’s open questions, most participants were able to remember at least some aspects of the early stages of their deterioration as the interview progressed. Use of a written timeline appeared to be helpful for some, whilst others employed their own recall strategies. A number of challenges to recall were outlined, such as the presence of residual psychotic symptoms, cognitive difficulties or a “sealing over” recovery style. The speed of deterioration affected identification of early signs, with a very abrupt or very gradual onset being particularly challenging.

Having identified the probable onset of relapse, there were often substantial barriers to seeking help from services. The infrequency of appointments with clinicians hindered early help seeking for some, whilst others found their efforts to directly access help were blocked, despite having experienced a clear deterioration in their mental health. A family or friend confidant was an important means of gaining assistance, although the supportive presence of significant others was not always available.

### Clinical recommendations

Given the considerable variation in participants’ experiences and circumstances, it is clear that early signs interventions need to be individually tailored. We have made several clinical recommendations, which may be summarised as follows:

•When timing the intervention, weigh up level of insight versus ability to remember.

•Flexibly use strategies, including the client’s own, to prompt memories.

•Be aware of potential challenges (e.g. social isolation, avoidant attachment style, high familial expressed emotion, sealing over recovery style) and work to overcome these in the intervention.

•Therapeutically target experiences that are both early signs and accelerants of relapse (e.g. emotional response to life events, feeling overwhelmed by anomalous experiences, searching for meaning).

•Where appropriate, emphasise medication adherence and stress reduction when relapse risk factors are present.

•Use early signs interventions to increase service users’ self-efficacy and sense of control (e.g. when reducing medication).

•Respond promptly and pro-actively to help seeking.

### Limitations

Although the sample size was larger than other qualitative research in this area, caution should be used when attempting to generalise findings to the whole psychosis population. The sample was diverse in terms of a number of demographic and clinical variables but certain groups may have been under-represented. A number of eligible service users declined to take part in the research (exact number unknown) and it is difficult to comment on the effect this may have had. As has been mentioned, in line with the study design, all participants had relapsed within the past six months which may have biased some of the conclusions (e.g. regarding barriers to help seeking). For practical reasons, none were out of contact with services. Finally, the study was conducted in English NHS services, so some of the results may only be applicable in this context.

Interviews were conducted and coded by a non-clinical researcher (EE), though the rest of the team had considerable experience of CMHTs and other clinical services. Since the aim of the research was to inform a specific type of intervention, this influenced the conduct and analysis of the interviews.

Unlike other studies, we did not interview participants’ family or carers, although the results suggested that they often have an important role. Though we planned to do so only seven participants had high levels of contact with these individuals and, of these, some were unwilling for them to be approached. This limited our study to a particular perspective, if that of the critical group in terms of targeting relapse prevention interventions.

### Future research

Firstly, a prospective investigation of the following putative early signs of relapse would be valuable: feeling overwhelmed by experiences; searching for meaning in experiences; subjective anomalous (basic symptom like) experiences. Secondly, the role of self-efficacy and other possible mediators of avoidant or maladaptive coping strategies in this process could be investigated. Thirdly, it would be worth prospectively comparing coping strategies of relapsers and non-relapsers in order to further examine effective coping strategies, reduce the skew towards ineffective relationships with services and eliminate distortions due to retrospective investigation. Fourthly, further investigation of the help seeking process in relapse is warranted, for example regarding the role of attachment style, and service related barriers to help seeking. Finally, given that a number of recommendations have been made in the current study, it would be valuable to incorporate them into an intervention and test its effectiveness in a randomised controlled trial.

## Abbreviations

NHS: National health service; DSM-IV: Diagnostic and statistical manual of mental disorders (fourth edition); ICD-10: International statistical classification of diseases (tenth revision); CPN: Community psychiatric nurse; CMHT: Community mental health team.

## Competing interests

The authors declare that they have no competing interests.

## Authors’ contributions

EE designed the study, conducted and coded the interviews and wrote the paper. CB and RD provided overall supervision of the study, assisted with analysis of the data, and contributed to drafts of the paper. FL provided consultation in all aspects of the qualitative design and analysis and commented on drafts of the paper. All authors read and approved the final manuscript.

## Pre-publication history

The pre-publication history for this paper can be accessed here:

http://www.biomedcentral.com/1471-244X/14/201/prepub
